# Material aspects of sintering of EAC-1A lunar regolith simulant

**DOI:** 10.1038/s41598-023-50391-y

**Published:** 2023-12-27

**Authors:** Juan-Carlos Ginés-Palomares, Miranda Fateri, Tim Schubert, Lilou de Peindray d’Ambelle, Sebastian Simon, Gregor J. G. Gluth, Jens Günster, Andrea Zocca

**Affiliations:** 1 Faculty of Mechanical Engineering and Materials Science, Aalen University, Beethovenstraße 1, 73430 Aalen, Germany; 2 Materials Research Institute Aalen, Aalen University, Beethovenstraße 1, 73430 Aalen, Germany; 3 Division 5.4 Advanced Multi-materials Processing, Bundesanstalt für Materialforschung und -prüfung (BAM), Unter den Eichen 87, 12205 Berlin, Germany; 4 Division 7.4 Technology of Construction Materials, Bundesanstalt für Materialforschung und -prüfung (BAM), Unter den Eichen 87, 12205 Berlin, Germany

**Keywords:** Ceramics, Aerospace engineering, Mechanical properties

## Abstract

Future lunar exploration will be based on in-situ resource utilization (ISRU) techniques. The most abundant raw material on the Moon is lunar regolith, which, however, is very scarce on Earth, making the study of simulants a necessity. The objective of this study is to characterize and investigate the sintering behavior of EAC-1A lunar regolith simulant. The characterization of the simulant included the determination of the phase assemblage, characteristic temperatures determination and water content analysis. The results are discussed in the context of sintering experiments of EAC-1A simulant, which showed that the material can be sintered to a relative density close to 90%, but only within a very narrow range of temperatures (20–30 °C). Sintering experiments were performed for sieved and unsieved, as well as for dried and non-dried specimens of EAC-1A. In addition, an analysis of the densification and mechanical properties of the sintered specimens was done. The sintering experiments at different temperatures showed that the finest fraction of sieved simulant can reach a higher maximum sintering temperature, and consequently a higher densification and biaxial strength. The non-dried powder exhibited higher densification and biaxial strength after sintering compared to the dried specimen. This difference was explained with a higher green density of the non-dried powder during pressing, rather than due to an actual influence on the sintering mechanism. Nevertheless, drying the powder prior to sintering is important to avoid the overestimation of the strength of specimens to be fabricated on the Moon.

## Introduction

In recent years there has been a renewed interest in lunar research and exploration, mainly led by the decision of National Aeronautics and Space Administration (NASA) to send astronauts to the Moon in the near future, as part of the space program Artemis^1^. In order to enable lunar exploration in a sustainable manner and for a prolonged time, it is necessary to develop technologies that allow the use of lunar resources in-situ. This requires the study and use of lunar regolith as raw material. However, due to the scarcity of lunar regolith on Earth, different lunar simulants have been developed. According to their composition, two groups of lunar simulants can be distinguished. The first group of simulants is formed by those materials used to replicate the regolith of the lunar highlands, which are rich in anorthite (plagioclase feldspar). The following simulants, among others, belong to this group**:** LHS-1 by the University of Florida^2^, NU-LHT-2M by the United States Geological Survey^3^ and OB-1 by the University of New Brunswick^4^. The second group replicates the lunar mare basalt regolith, which are rich in pyroxene and contain also plagioclase, ilmenite and olivine. For this purpose, the following simulants have been developed: JSC-1 by NASA Johnson Center/Orbitech^5^, JSC-2 by NASA/Zybeck^6^, BP-1 by NASA Kennedy Space Center^7^ and LMS-1 by the University of Florida^8^, among others.

Most of the above-mentioned simulants are not available in large quantities. Recently, the European Astronaut Center (EAC) in Germany, has developed EAC-1A which is a mare regolith simulant for use in the European Lunar Exploration Laboratory (LUNA) as a large-quantity material available for lunar research^9^. EAC-1A is a basanitic sand obtained from the volcanic area of Siebengebirge in Germany. In the study carried out by Engelschiøn et al., the chemical composition of the simulant was evaluated giving a result of 43.7% in SiO_2_, 11.9% MgO, 4.2% % (Na_2_O + K_2_O) and 2.4% TiO_2_ (other oxides not reported, i.e., reported total < 100%), which the authors consider to be comparable to the Apollo 17 samples^10^.

A recent review by Farries et al. highlights that sintering and melting are considered two of the most promising strategies to consolidate lunar regolith and utilize it as raw material, since only a source of energy without additional raw materials is needed^11^. In a sintering process the material is densified by mechanisms of atomic diffusion, without complete melting. The sintering and melting behavior of lunar simulants can however vary significantly, depending on the simulant type (mare or highland), mineralogical composition and content of glass^11^. The radiant furnace sintering of several simulants has been investigated in the literature, including CLRS-1^12^, CLRS-2^13^, ALRS-1^14^ , JSC-1A^15^, DNA^15^ and our own study on JSC-2A^16^.

So far, a characterization of the sintering and melting behavior of EAC-1A is not available in the literature, despite it being one of the few simulants available for large-scale experiments. In this work, EAC-1A is characterized with focus on its sintering behavior in a radiant furnace.

## Results

### Particle size distribution and scanning electron microscopy

**Figure 1:**
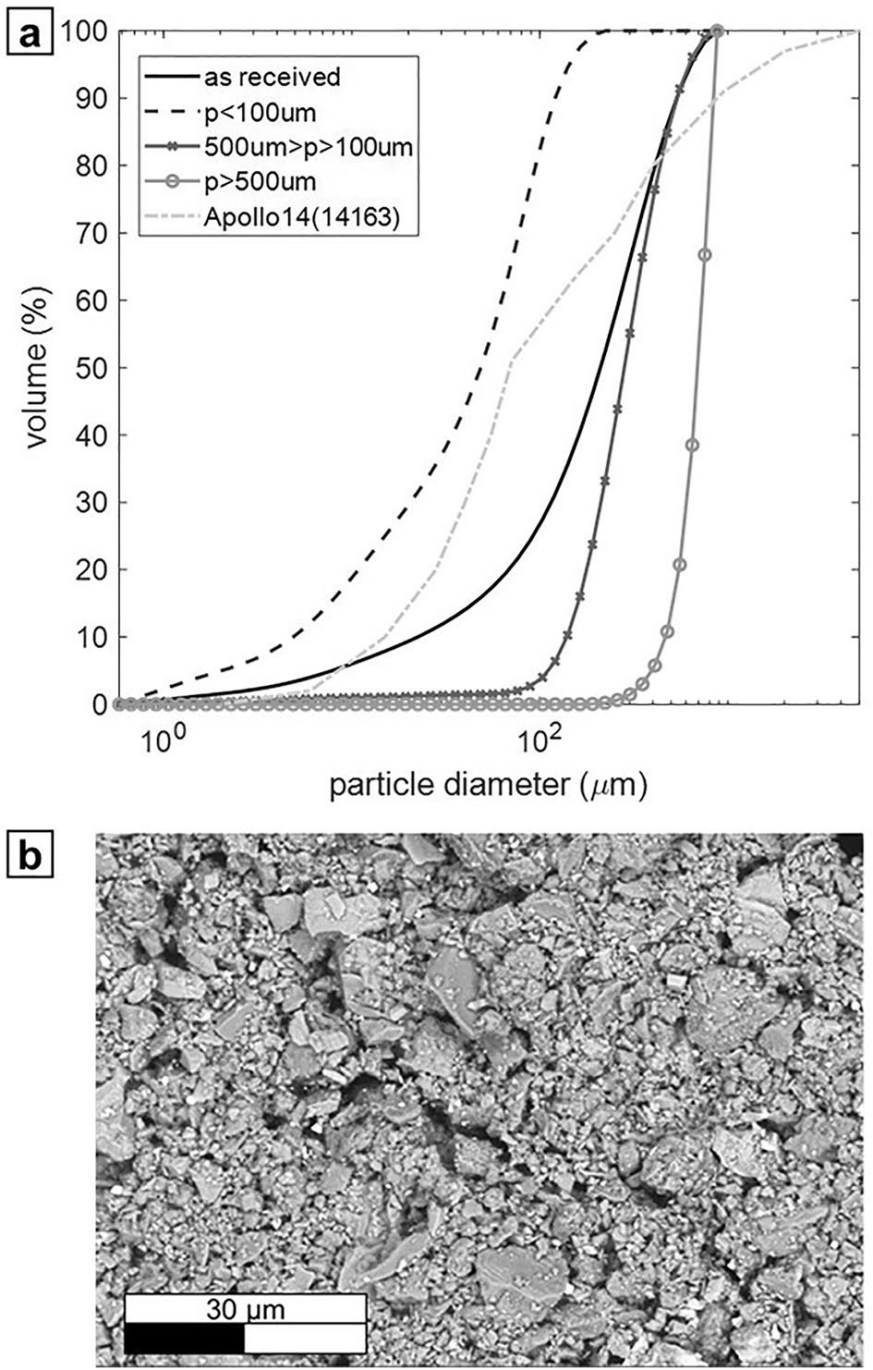
(**a**) Particle size distribution of the EAC-1A samples and Apollo 14 regolith’s sample^17^. (**b**) SEM image of EAC-1A powder.

The particle size distribution (PSD) determinations were performed for EAC-1A as received and after sieving: above a 500 µm sieve (*P* > 500 µm), between 100 µm and 500 µm sieves (500 µm > *P* > 100 µm), and under a 100 µm sieve (*P* < 100 µm). The PSD results are presented in Fig. 1 and compared to 14,163-regolith sample from the Apollo 14 mission^17^. The powder as received had a measured *D*(v,0.1) = 22 µm, *D*(v,0.5) = 210 µm and *D*(v,0.9) = 535 µm.

After sieving the material, PSD measurements were repeated for each sieved fraction. For particles larger than 500 µm, the particle size distribution is the following: *D*(v,0.1) = 468 µm, *D*(v,0.5) = 694 µm and *D*(v,0.9) = 840 µm. In the case of particles in the range of 500 µm to 100 µm, the PSD is *D*(v,0.1) = 139 µm, *D*(v,0.5) = 282 µm and *D*(v,0.9) = 536 µm. Finally, for grains smaller than 100 µm, *D*(v, 0.1) = 5 µm, *D*(v,0.5) = 49 µm and (v,0.9) = 121 µm.

The PSD of Apollo 14 sample has the following values: *D*(v,0.1) = 15 µm, *D*(v, 0.5) = 75 µm and *D*(v,0.9) = 900 µm. Comparing the EAC-1A as received with the regolith sample, it is noticeable that the mean value of the PSD is larger for EAC-1A (210 µm). Additionally, the Apollo sample of lunar regolith presents a higher content of particles between 20 and 200 µm, but also a significant amount of very large grains up to 5 mm in size. In the case of very fine particles, however, both samples show a similar PSD (*D*(v,0.1) of 22 µm for the EAC-1A and 15 µm for the lunar regolith).

Figure 1b shows the scanning electron microscopy (SEM) image of a sample of the as-received EAC-1A lunar simulant powder, revealing that the simulant powder is made of sharp-edged particles with irregular shapes. Due to the magnification chosen to visualize the smaller particles, only particles up to approximately 20 µm are visible in Fig. 1b.

### X-ray diffraction

**Figure 2:**
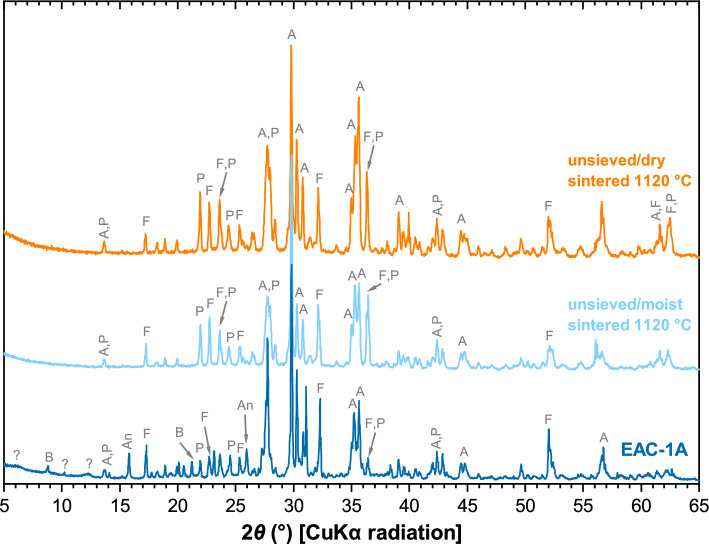
XRD patterns of as-received EAC-1A (bottom), the unsieved/moist powder sintered at 1120 °C (middle), and the unsieved/dry powder sintered at 1120 °C (top). Major peaks are labelled: B, biotite; A, diopside/augite; P, plagioclase; F, forsterite; An, analcime; ?, unidentified mineral(s)-likely clay mineral(s) and/or zeolite.

The mineralogical composition of the EAC-1A material and some of the sintered materials (see below) was studied by X-ray diffraction (XRD). The XRD pattern of EAC-1A is shown in Fig. 2. Forsterite, plagioclase, diopside/augite and biotite were found to be the major constituents of the material. In addition, minor amounts of analcime and ilmenite were identified. Broad reflections at *d* = 14.4 Å (2*θ* = 6.0°) and *d* = 7.2 Å (2*θ* = 6.0°), and a peak at *d* = 8.7 Å (2*θ* = 10.2°) could not be unequivocally assigned to a specific mineral; these reflections are attributed to alteration products such as clay minerals (vermiculite, smectite, or chlorite) and/or zeolites (e.g., faujasite) in the material.

In addition to the measurements for phase identification, XRD measurements for Rietveld quantitative phase analysis (RQPA) of EAC-1A were performed (see Supplementary Figure S1). The minor or trace amount of ilmenite as well as the unidentified clay/zeolite minerals were not included in the analysis. The results of the RQPA are compared with the results of Schleppi et al*.*^18^ for EAC-1A in Table 1 and will be further discussed below.

**Table 1:** Results of the RQPA of EAC-1A, compared with the results of Schleppi et al.

Mineral	Composition	Abundance (%)	Abundance according to Schleppi et al.^18^ (%)
Plagioclase	(Ca,Na)(Al,Si)_4_O_8_	28.3	27.2^b^
Diopside/augite	(Ca,Na)(Mg,Fe,Al,Ti)(Si,Al)_2_O_6_	28.4	35.5^c^
Forsterite	(Mg,Fe)_2_SiO_4_	29.3	13.3
Biotite	K(Mg,Fe^2+^,Al)_3_(Al,Si)_4_O_10_(OH,F)_2_	11.3	7.6^d^
Unidentified mineral^a^		n.a	
Analcime	NaAlSi_2_O_6_·H_2_O	2.9	–
Ilmenite	FeTiO_3_	Minor/trace	2.0^e^
Glass		–	14.4

^a^Unidentified mineral(s) at *d* = 14.4 Å (2*θ* = 6.1°), *d* = 8.7 Å (2*θ* = 10.2°) and *d* = 7.2 Å (2*θ* = 6.0°); likely clay mineral(s) and/or zeolite.

^b^Feldspars combined; viz., plagioclase (Ca,Na)(Al,Si)_4_O_8_: 13.8%, and K-feldspar KAlSi_3_O_8_: 13.4%.

^c^Pyroxenes combined, including diopside, augite etc.

^d^Mica/biotite and “alteration” combined, including annite KFe^2+^_3_AlSi_3_O_10_(OH,F)_2_, phlogopite KMg_3_AlSi_3_O_10_(F,OH)_2_, and chlorite.

^e^“Oxide minerals”, including ilmenite.

### Karl–Fischer titration

The water content of the sieved and unsieved samples of the regolith simulant EAC-1A was determined by Karl-Fischer titration technique. The Karl-Fischer titration results revealed a water content of 1.21% in the as-received EAC-1A sample. The results for the sieved samples indicated an increase of water content with decreasing particle size: 0.77% for *P* > 500 µm, 0.99% for 500 µm > *P* > 100 µm, and 1.31% for *P* < 100 µm.

### Thermogravimetric analysis and differential scanning calorimetry

**Figure 3:**
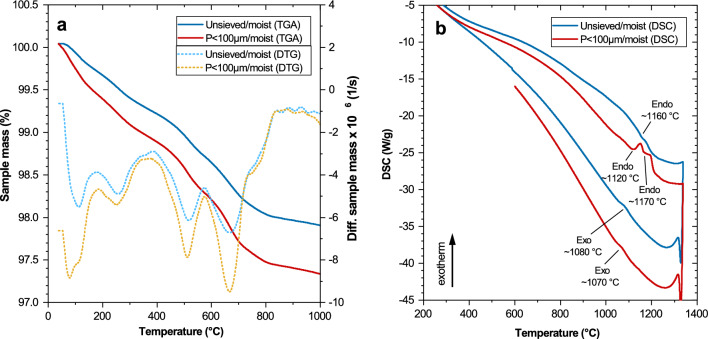
TGA and DTG curves (**a**) and DSC curves (**b**) of EAC-1A unsieved/moist and the fraction P < 100 µm/moist.

The results of the thermogravimetric analyses (TGA) of EAC-1A and the fraction *P* < 100 µm in the temperature range 40–1000 °C, including the derivative thermogravimetry (DTG) curves, are shown in Fig. 3a. The TGA curves exhibited distinct mass loss steps at temperatures around 100, 250, 500, and 650 °C. The total mass loss after heating to 1000 °C was 2.1% for the unsieved material and 2.7% for the *P* < 100 µm fraction.

The mass losses at around 100 °C are assigned to loss of physically bound (*i.e*., adsorbed) molecular water. The slightly higher total mass loss of the *P* < 100 µm fraction, compared to the unsieved powder, was mainly caused by a higher mass loss in this temperature range, *i.e*., a higher amount of adsorbed water, due to its higher specific surface area. The mass losses around 250 °C are assigned to the loss of zeolitic water from analcime^19,20^. The two steps around 500 and 650 °C, respectively, may be related to the dehydration/dehydroxylation of the unidentified clay mineral(s) or zeolite in the sample; for example, some chlorites and smectites exhibit a prominent TGA/DTG signal at ~ 500 °C and/or ~ 680 °C^21–23^. In addition, depending on its composition, biotite can exhibit a minor mass loss in the range 500–700 °C^24^, which might have contributed to one or both of these steps. Instead of, or in addition to, release of water from clay minerals or zeolites, the two signals at 500 and 650 °C may be caused by the decomposition of carbonates (*cf*. the infrared spectroscopy results below) that were not detected by XRD, either because they were amorphous/poorly crystalline^25^ or because they were present in a too low amount. The latter assignment provides an explanation, at least partially, for the difference between the total mass loss determined by TGA and the total water content determined by Karl-Fischer titration.

The DSC curve Fig. 3b of the as-received EAC-1A displayed an endothermic peak on heating at ~ 1160 °C and an exothermic peak on cooling at ~ 1080 °C. The peak at 1160 °C is related to melting of the material, while the peak during cooling at 1080 °C is assigned to the crystallization of the melt. (The feature at ~ 1320 °C is an artefact of the measurements.) It is notable that only one prominent endotherm was observed in the DSC curve on heating, i.e., no additional signals at lower temperatures could be clearly distinguished. While the endotherm at 1160 °C reflects the melting of the crystalline phases of EAC-1A, a significant fraction of glass in the bulk material would be expected to cause a signal at a lower temperature. The fact that no such peak was observed is in line with the XRD results, which showed that no or only an insignificant fraction of glass was present in the material. However, for the *P* < 100 µm fraction, two endothermic features (at ~ 1120 and ~ 1170 °C) where observed in the heating curves (Fig. 3b). This observation might indicate either that this fine fraction contains some glassy phase or that different crystalline minerals in the material start to melt at slightly different temperatures. The latter possibility is supported by the SEM–EDS results (see below), which suggest that a partially molten plagioclase matrix forms and surrounds other phases during sintering EAC-1A.

The mass losses found in the TGA/DTG curves at 500 and 650 °C could not be clearly discerned as endothermic peaks in the respective DSC curves. This is presumably due to the limited sensitivity of the DSC measurements, which does not allow to identify these expected small peaks, each representing a mass loss of only ~ 0.4%, against the background noise.

### Infrared spectroscopy

**Figure 4:**
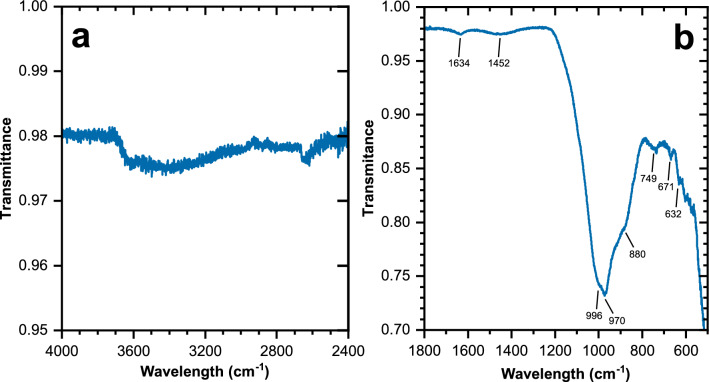
ATR-FTIR spectrum of EAC-1A (as received); 4000–2400 cm^−1^ range (**a**) and 1800–500 cm^−1^ range (**b**).

The infrared spectrum of EAC-1A, obtained by ATR-FTIR spectroscopy, is shown in Fig. 4. The prominent band at ~ 1000–970 cm^−1^, extending to approx. 1200 cm^−1^ at higher wavenumbers, and the shoulder at ~ 880 cm^−1^ are assigned to the Si–O–T (T = Al, Si) stretching vibrations of the aluminosilicate minerals in the material. The broad band in the range 3700–2900 cm^−1^ is caused by O–H stretching of OH groups as well as molecular water, while the band at ~ 1635 cm^−1^ is due to H–O–H bending of molecular water. The band at 3700–2900 cm^−1^ is rather featureless, i.e., vibrations of specific OH groups in the biotite^24,26^ cannot be discriminated, presumably due to the presence of other minerals containing OH groups and/or water, such as analcime and the alteration products inferred from XRD analysis (Table 2), which cause broad bands in the same wavenumber range^20,21^. The band around 1460 cm^−1^ is attributed to carbonate ions in carbonate minerals, supporting the partial assignment of the mass loss steps at ~ 500 and ~ 650 °C in the TGA to such compounds.

### X-ray fluorescence analysis

The chemical composition of EAC-1A is shown in Supplementary Table S1 online. The measured composition, reported in terms of equivalent oxides, was similar to the results in Engelschiøn et al.^10^. The concentration of SiO_2_ in our measurements was very close at 43.7%. The concentration of TiO_2_ (2.1%) and the sum of Na_2_O + K_2_O (2.8% + 1.0%) was also comparable. The small difference in MgO (14.3% in our work, 11.9% in^10^) may be due to variations between batches.

The X-ray fluorescence analysis measurement repeated on the simulant after heat treatment at 900 °C in vacuum (Supplementary Table S1) reveals that the composition is almost unchanged after the heat treatment. Interestingly, the sum of concentrations is close to 100% after heat treatment, but only 96.6% in the as-received material. This result suggests that the as-received material contains elements that are not detected by XRF, which could be eliminated after heat treatment. Typically, these elements can be hydrogen in water (adsorbed or chemically bound) and carbon in carbonates (Ca and Mg carbonates). This is consistent with the Karl-Fischer titration, the TGA/DTG and with the ATR-FTIR results. Furthermore, it suggests that a heat treatment at 900 °C in vacuum can eliminate these compounds, which are practically absent in lunar regolith and thus not representative of it^27^, before the simulant starts sintering.

### Hot stage microscopy

The parameters obtained with Hot Stage Microscopy (HSM) are the relative changes in specimen’s area and shape factor while the specimen is heated. The specimen’s area is calculated in the software as the number of pixels forming the silhouette of the specimen, and its relative variation is expressed as percentage of the initial area. The shape factor is the ratio between the width and the height of the silhouette. The results of these analysis are shown in Supplementary Fig. S2.

The characteristic temperatures obtained with the instrument for each particle size fraction of EAC-1A are presented in Table 2. The experiments were performed in air atmosphere and in argon.

**Table 2:** Temperatures (°C) obtained for each particle size, average value for each experiment, under air and argon, respectively.

	Air			Argon		
	As received	500 µm > *P* > 100 µm	*P* < 100 µm	As received	500 µm > *P* > 100 µm	*P* < 100 µm
SST (°C)	1144 ± 3	1147 ± 4	1125 ± 3	1094 ± 4	1083 ± 8	1073 ± 4
DT (°C)	1167 ± 21	1158 ± 27	1171 ± 3	1131 ± 15	1115 ± 12	1134 ± 2
ST (°C)	1195 ± 1	1192 ± 0	1189 ± 1	1161 ± 3	1153 ± 3	1148 ± 1
HT (°C)	1204 ± 0	1199 ± 1	1197 ± 1	1177 ± 2	1165 ± 2	1165 ± 1
FT (°C)	1324 ± 13	1281 ± 7	1308 ± 17	1362 ± 8	1303 ± 58	1350 ± 33

The results are expressed as the temperature ± 1 standard deviation (three measurements).

The characteristic temperatures during the thermal cycle are categorized as follows^6^:

- Start of sintering temperature (SST): the temperature at which the pressed specimen starts to shrink.
- Softening temperature (DT): the temperature at which rounding of the corners of the specimen’s silhouette is observed.
- Sphere temperature (ST): the temperature at which the silhouette of the specimen forms a circle.
- Hemisphere temperature (HT): the temperature at which the silhouette of the specimen forms a semicircle.
- Flow temperature (FT): the temperature at which the minimum height of the molten material occurs.

Sintering in air started between 1125 °C and 1150 °C. At ~ 1170 °C the specimens started to soften, and at ~ 1200 °C melting occurred.

When comparing the results obtained from the air and argon experiments, small differences in the temperature values of the specimens are noticed. The experiments performed with argon showed lower temperature values for each fraction and temperature analysed, except in the case of the flow temperature. In particular, the start of sintering temperature was approximately 50 °C lower in argon than in air. In most cases, the characteristic temperatures decreased with deceasing particle size, *i.e*., the characteristic temperatures of the *P* < 100 µm fraction were generally lower than for the as-received EAC-1A powder. This is in accord with the DSC data, showing only one endotherm for the unsieved EAC-1A material and two endotherms for the *P* < 100 µm fraction, with the first endotherm found at slightly lower temperature than the endotherm of the unsieved material. The results of flow temperature should however be interpreted carefully, since the measured values had a comparatively large variation between three specimens (standard deviations given in Table 2).

### Pressing and sintering of EAC-1A lunar regolith simulant

Four sets of specimens are compared to investigate the effect of the particle size and of pre-drying:

1. As-received, i.e. unsieved and not dried (unsieved/moist)
2. Unsieved, dried at 200 °C for 48 h (unsieved/dry*)*
3. Sieved *P* < 100 µm, not dried (P < 100 µm/moist)
4. Sieved *P* < 100 µm, dried at 200 °C for 48 h (P < 100 µm/dry)

Tablets were pressed in a hydraulic press with a 16 mm round die, in two steps at 6 MPa for 10 s and at 31 MPa for 20 s. No binder was used except for the unsieved/dry powder, for which a drop of isopropanol had to be added to obtain stable tablets.

The geometrical density of the pressed specimens was:

1. 2.26 ± 0.01 g/cm^3^ for unsieved/moist specimens
2. 2.20 ± 0.02 g/cm^3^ for unsieved/dry specimens
3. 2.14 ± 0.02 g/cm^3^ for P < 100 µm/moist specimens
4. 2.08 ± 0.01 g/cm^3^ for P < 100 µm/dry specimens

The sintering of the EAC-1A pressed tablets was investigated under vacuum (10^−3^ mbar). All specimen groups were heated at a constant ramp of 10 °C/min up to the final temperature, with a 1 h hold time. The final temperature was varied in the range 1100–1160 °C. The temperature range was suggested by the results of the HSM measurements in argon: 1100 °C is slightly above the measured start of sintering temperature; 1160 °C is close to the sphere temperature, the temperature at which the material melts and sintering is no longer possible.

For the P < 100 µm/dry fraction, the material sinters until 1140 °C without significant deformations or defects. The P < 100 µm/moist tablets present small bubbles at 1140 °C (Supplementary Fig. S3), which possibly indicate the release of gases at slightly lower temperature compared to the dry material.

At 1160 °C, melting occurs for both the dried and moist material: the tablets are stuck on the ceramic base and large bubbles, as well as deformations, are noticed on the surface (Supplementary Fig. S4).

The unsieved/dry material instead already started to significantly deform at 1140 °C and the tablets present large bubbles on the top (Supplementary Fig. S5). It is noticed that the unsieved/moist simulant does not melt significantly at 1140 °C but presents small bubbles on its surface. According to these results, the *P* < 100 µm fraction can be sintered at slightly higher temperature compared to the unsieved simulant. The pressed tablets before sintering had a light grey aspect and some darker and lighter grains were observable (Supplementary Fig. S6). When sintered, the tablets became darker.

The XRD patterns of the unsieved/dry and the unsieved/moist material after sintering at 1120 °C are included in Fig. 2. Comparison with the pattern of the as-received EAC-1A shows that biotite, analcime and the alteration products (clay mineral(s)/zeolite) were no longer present after the heat treatment, i.e., these phases had fully decomposed and likely released iron. The darkening of the material during sintering can thus be explained by the oxidation of ferrous iron (Fe^2+^) in the biotite^24,26^ and the alteration products during heating and decomposition. In addition, iron oxidation on the surface of forsterite appears possible^28^, but would be expected to occur only to a minor extent.

### Sintering densification

**Figure 5:**
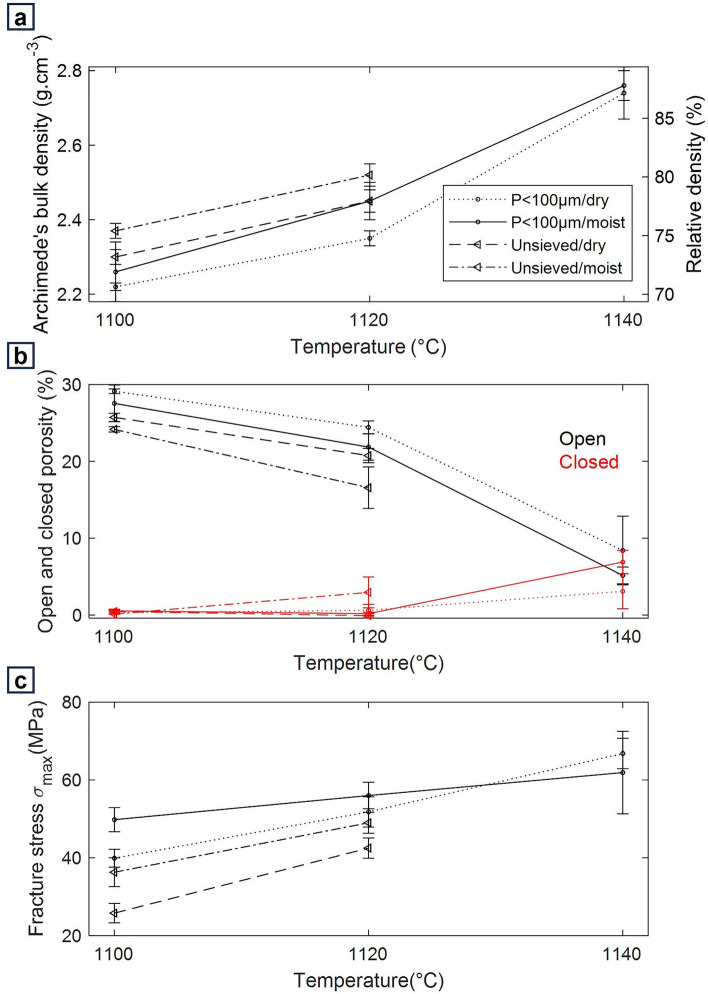
(**a**) Densities of the specimens after sintering (Archimedes method), (**b**) open and closed porosities of the specimens after sintering and (**c**) ball-on-three-balls mechanical tests results for the EAC-1A sintered tablets.

Figure 5a shows the density of the pressed tablets after sintering measured by the Archimedes’ method. The theoretical (true) material density was measured by helium pycnometry. For the as-received simulant, the material partially melted at 1140 °C and thus its density could not be determined.

The unsieved simulant sinters and reaches a maximum density around 2.5 g/cm^3^ at 1120 °C. For the finer fraction, it is possible to reach a final density around 2.7 g/cm^3^ since a higher sintering temperature of 1140 °C can be used before observing bubbles forming in the material.

At temperatures higher than 1120 °C however the shrinkage and the properties of the specimens become very sensitive to small variations due, e.g., to positioning in the oven chamber which can lead to temperature differences of ± 5 °C. Gas is also released from the material and pores can be formed. Figure 5b shows that until 1120 °C there is sufficient open porosity for the gases to be released. At temperatures higher than 1120 °C the open porosity decreases with an increase of closed porosity. At this point, gas can be trapped in the specimen forming bubbles. These factors contribute to variations between specimens, leading to the large standard deviation in the measurements.

A general trend is observable: the moist powders (P < 100 µm/moist and unsieved/moist) show higher densities before and after shrinkage compared to the corresponding dried powders. This phenomenon is most likely related to the pressing step and not due to a difference in sintering behaviour. Adsorbed water can act as a lubricant and as a binder during pressing, leading to a higher pressed density. It can be observed that the densification curves are almost parallel between 1100 and 1200 °C, suggesting that the sintering mechanism and the densification kinetics are not influenced by the initial humidity in the material.

Interestingly, the initial slope (1110 °C to 1120 °C) of the densification curves in Fig. 5a is also very similar between the unsieved and the *P* < 100 µm simulant. This behaviour is generally not expected, since smaller particles have higher sinter activity, as also confirmed by the lower start of sintering temperature in Table 2. However, it should be noticed that the bulk density of specimens pressed from the unsieved powder is significantly higher (t-test, *α* < 0.05) compared to the *P* < 100 µm fraction (*p*-value = 3.67 × 10^–8^ for dry specimens and p-value = 7.35 × 10^–14^ for moist specimens, respectively). As shown in Fig. 1, the unsieved simulant’s PSD is a lot broader than that of the finer fraction. The smaller particles in the unsieved powder can fill the gaps between coarser particles, and consequently achieve a higher packing. Possibly, the higher number of contacts between neighbouring particles, in the unsieved powder, offsets the higher sintering activity of the finer particles, at least in the early stage of sintering.

### SEM–EDS analysis of microstructural evolution during sintering

**Figure 6:**
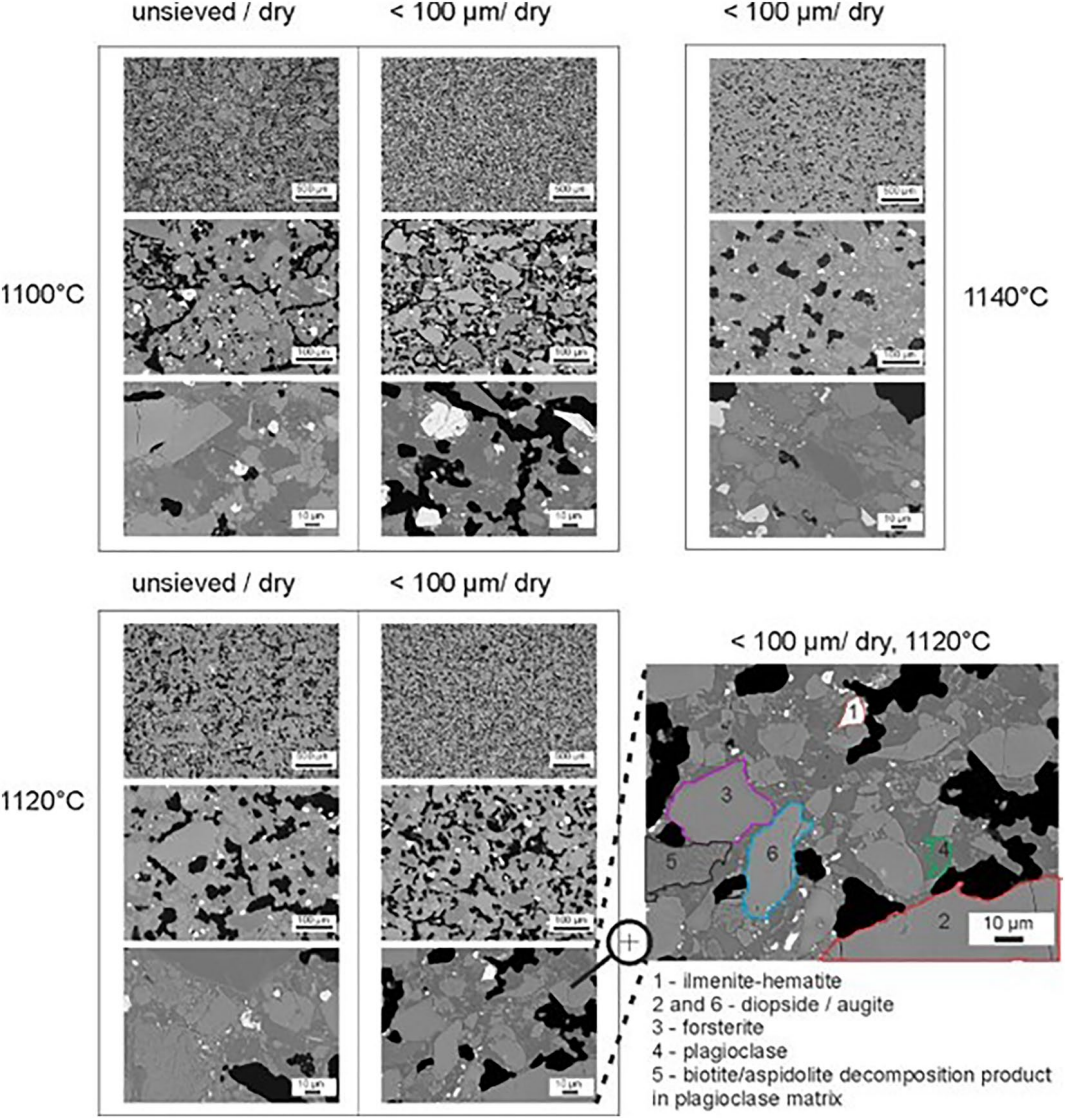
SEM–EDS analysis of the microstructural evolution of EAC-1A during sintering.

The microstructural evolution of EAC-1A during sintering in vacuum (10^−3^ mbar) between 1100 and 1140 °C (1 h holding time) is shown in Fig. 6. Consistent with the results of Table 2 (in argon) and Fig. 5a it can be observed that sintering is at the initial stage around 1100 °C, with neck formation between grains and a microstructure characterized by interconnected porosity. Sintering proceeds in a narrow range of temperature, with an increased densification at 1120 °C. The SEM observations confirm that necking and densification is achieved for both fractions unsieved/dry and P < 100 µm/dry. However, it can be visually observed that the microstructure of the sintered unsieved/dry material is less homogeneous (especially at 1100 °C), comprising better densified areas and large pores. Formation of a significant fraction of closed porosity, which indicates an advanced sintering stage, can be clearly observed only in the P < 100 µm specimen at 1140 °C. This result is consistent with the measurements of open and closed porosity reported in Fig. 5b.

EDS analysis (see Fig. 6 and Supplementary Fig. S7) combined with the XRD results (Table 1) supported the identification of the microstructural phase evolution during sintering. The bright grains in the SEM images (area “1”) can be identified as ilmenite-hematite. Large grains of diopside/augite (e.g., “2” and “6”) are also found. Additionally, forsterite grains can also be identified, such as area “3”. Plagioclase (the darker grey phase, e.g., “4”) forms an interconnected matrix in which the other phases are embedded. Likely, this microstructure is formed after (possibly partial) melting of the plagioclase phase. Areas with finely distributed grain fragments are also visible (“5”). These can most likely be assigned to biotite decomposition products in a plagioclase matrix. Interestingly, this phase is poor in potassium and rich in sodium, compared to ‘stoichiometric’ biotite. This suggests that the decomposed phase may have contained aspidolite, i.e., the sodium analogue of the magnesium-rich biotite end-member phlogopite. As mica species are difficult to unambiguously identify, this phase is labelled as biotite/aspilodite decomposition product.

### Mechanical characterization

For mechanical testing, the ball-on-three-balls method was used to measure the biaxial strength of sintered specimens. Figure 5c reports the biaxial strength (σ_max_) of tablets for each sintering temperature and for each powder under study. The load–displacement curves of the tablets can be found in Supplementary Fig. S8.

The biaxial strength as expected follows the same trend of increasing strength with increasing densification. It can be observed that the strength of tablets produced with the finer fraction tends to be slightly higher than that of the unsieved material. Most likely this is due to the finer microstructure of the material and to the higher number of formed sintering necks.

## Discussion

A series of characterization methods have been applied to investigate the properties of the lunar regolith simulant EAC-1A. The characteristics of lunar regolith are difficult to recreate with terrestrial material, particularly considering the unique features of lunar regolith^29^. The content of different minerals can play a role in the melting and sintering properties and can be compared with the composition of other lunar simulants. In the present study, the four most abundant minerals found in EAC-1A (viz., forsterite, plagioclase, diopside/augite and biotite) comprised ~ 97% of the material, ignoring the trace ilmenite and the unidentified clay/zeolite alteration product (Table 2). The present phase analysis of EAC-1A agrees qualitatively with the analysis of Engelschiøn et al.^10^, who also identified forsterite, Ca-plagioclase (anorthite), diopside and augite. An apparent difference is that Engelschiøn et al. identified two different olivine minerals, namely forsterite (nominally Mg_2_SiO_4_) and fayalite (nominally Fe_2_SiO_4_), in the simulant, while forsterite was identified as the only olivine in our work. This, however, does not necessarily imply a significant difference between the iron contents of the olivine in the two studies, as forsterite and fayalite form a complete solid solution [(Mg,Fe)_2_SiO_4_] and their iron contents were not determined in the two studies. A major difference between the present work and the study of Schleppi et al.^18^ is that in their XRD analysis a considerable amount of glass (~ 14%) was found in EAC-1A, while in our study the material was found to be virtually fully crystalline. This latter result is consistent with our DSC measurement (Fig. 3b) and with the DSC analysis of Engelschiøn et al., in which no glass crystallization peaks are visible^10^. Generally, the composition of actual lunar rocks can be fully crystalline or comprising a considerable amount of glass. The glass present in lunar regolith can derive from vulcanism or from the melting of material due to meteorite impacts. Thus, considering the influence of the glass content in the simulant is important for the envisioned application, specifically for the sintering behaviour of the material in this work.

The sintering and melting temperatures of EAC-1A were investigated by hot stage microscopy. In the ceramic community, hot stage microscopy is an established technique to investigate the sintering behavior of ceramics and glasses^30^. Several studies^30,31^ describe HSM as a recognised standard method for the study of the thermal behaviour (mainly the sintering, softening and melting) of ceramic materials and glasses in both industry and academia. One of the main advantages compared to dilatometers (also widely used instruments for this scope) is that the specimen is not in contact with any external measuring element (e.g., dilatometer rod)^30^. Especially for materials that sinter by viscous flow or for which sintering involves the partial formation of a liquid phase (such as EAC-1A) there are no external loads that can influence the measurement. In these experiments, when the specimen reaches the hemi-sphere temperature (HT in Table 2), the material has already reached the liquid state. The values of HT obtained for the EAC-1A specimens in air can be compared to the results of the study on lunar regolith melting carried out by Akimoto et al. In their study, regolith sample 10,085 showed a melting temperature of around 1220 °C at 1 atm in oxygen^32^. The HT of EAC-1A in air shows similar values (~ 1200 °C) and is also consistent with the position of the melting peak in our DTA measurement, which is centered at 1192 °C. Furthermore, Table 1 shows a narrow range of temperatures from the start of sintering of the EAC-1A material to its hemi-sphere temperature (when the material is already flowing, and sintering is no longer possible) of about 60–80 °C. Sintering most likely starts concomitantly with the formation of a liquid phase and follows a liquid phase sintering mechanism. SEM–EDS analysis (Fig. 6) suggests that starting at approximately 1100 °C partial melting occurs, generating an interconnected plagioclase matrix in which grains of ilmenite-hematite, diopside/augite, forsterite and biotite/aspidolite decomposition products are embedded. As expected, the start of sintering slightly decreases for the finer particle size (around 20 °C lower for the sieved powder *P* < 100 µm than the simulant as received). The temperature at which the material is fully molten, considered here for comparison as the hemisphere temperature (HT), is instead similar for all considered particle size fractions. This indicates that the material composition is likely homogeneous, and that no significant separation between mineral phases results from sieving. Considering the fraction of EAC-1A unsieved, the reduction observed in the argon experiments is 52 °C for SST, 36 °C for DT, 34 °C for ST and 27 °C for HT. The observed small difference is likely due to oxidation of the material, which causes a slight increase of the melting temperature in air. This effect has been observed also with other simulant, see e.g. the work of Meurisse et al. on JSC-1A^15^. The sintering results in vacuum suggest that the sintering behavior of the material lies in between the HSM curves in air and argon. HSM results therefore can be helpful to support the optimization of the sintering parameters.

The study of EAC-1A allows us also to discuss the influence of the simulant mineral composition on its sintering behavior. In simulants with a large glass fraction, such as JSC-1A and JSC-2A, it is expected that the powder starts to sinter at lower temperature due to viscous flow (until the glassy phase is crystallized). Farries et al. for example conclude that simulants with a glass phase, as is present in true lunar regolith, sinter more uniformly and at lower temperatures than fully crystallized simulants^11^. Meurisse et al. compared the sintering of JSC-1A with DNA, the latter being a fully crystallized simulant^15^. They concluded, however, that the major influence on the sintering temperature is the composition of the plagioclase phase. DNA contains predominantly Na-rich plagioclase (albite), which has a lower melting temperature than Ca-rich plagioclase (anorthite), which is the predominant plagioclase phase in JSC-1A as well as in actual lunar regolith. EAC-1A is fully crystallized and contains plagioclase, which our XRD characterization indicated to be Ca-rich (anorthite yielded a better initial fit of the measured XRD patterns than albite); it is thus more suitable than albite-rich simulants such as DNA for a comparison of the effect of the glass phase. Interestingly, the optimal sintering temperature for EAC-1A determined in our current study is 1120 °C in vacuum, which is only slightly higher than the optimal temperature of 1100 °C suggested by Meurisse et al. for JSC-1A. An explanation for the minor effect of the glass phase on sintering is that most of the glass in JSC-1A crystallizes at temperatures much lower than those required for sintering. DSC studies revealed that most of the glass in JSC-1A crystallizes at 800 °C and a small remaining amount crystallizes at 1080 °C. Although a major effect of the glass content on the sintering behaviour of the simulant can be excluded, a minor influence cannot not be dismissed based on the experimental observations in this work. Indeed, our results suggest that EAC-1A sinters at slightly higher temperatures and reaches a lower final density compared to JSC-1A, although this might depend on small difference in the sintering conditions and the mineral composition of the simulant.

Another important feature to consider when evaluating the simulant is its water content. The determination of the water content in the powder was carried out by Karl-Fischer titration. The highest water content (1.3%) was found in the sample of the finest particle size (< 100 µm) and decreases when increasing the particle size of the sample, due to the reduction in specific surface area of the powder. The largest effect of the water content in the powder was on the pressed density of the specimens, while the sintering rate and mechanism does not seem to be influenced. The most likely explanation is that water acts as a lubricant and as a binder during pressing.

The interpretation of the results for a future application on the Moon should be treated with care, because actual lunar regolith (except in the shadow of craters at the lunar poles) has a water content close to zero. In particular, performing sintering experiments on a simulant without drying may lead to a significant overestimation (up to + 16% in our study) of the mechanical strength of components manufactured on the Moon. Therefore, for further studies it is recommended to carefully dry the simulant at 220 °C for 48 h. The biaxial strength achieved for the unsieved/dry material sintered in vacuum is approx. 40 MPa, which is in the range of strength of porcelain stoneware^33^. It can thus be envisioned that such sintered material could be used for ISRU in several applications ranging from stoneware to construction materials.

To complement this work and further characterize the sintered specimens described in this article, a detailed analysis of the microstructure of the sintered bodies will be carried out in a future study.

## Methods

### Particle size distribution

Particle size distribution (PSD) determination of the simulant was carried out using laser diffraction technique (Mastersizer 2000, Malvern Instruments). The powder was measured in dry conditions dispersed in air (air pressure 0.5–1 bar). The particle size distribution was calculated following the Fraunhofer diffraction method. A laser beam of 10 mm length and focal length of 300 mm was used. The as received EAC-1A simulant was sieved with the aim of obtaining three different particle sizes. The fractions selected were: *P* > 500 µm, 500 µm > *P* > 100 µm, *P* < 100 µm, where *P* refers to the particle size of the material.

### Scanning electron microscope

The morphology of the simulant was analyzed using a scanning electron microscope (SEM) (Sigma 300VP, Zeiss). For this investigation, the imaging conditions where as follows: Magnification 1000×, EHT = 10 kV, WD = 8.4 mm, Backscattered electron detector.

The microstructure of sintered EAC-1A tablets was investigated by SEM–EDS. The specimens were embedded in resin and prepared for microscope investigation. A polished surface was obtained on a grinding and polishing machine (Tegramin-30, Struers) by successive steps from 500 to 1200 grade grinding surface, followed by 9 µm, 3 µm, 1 µm and 0.25 µm diamond suspension polishing. The specimens were carbon coated in a sputtering chamber (CED 030, Baltec AG, Switzerland) and analyzed in a LEO Gemini 1530 VP SEM (Zeiss, Germany) mounting an EDX System XFlash Detector 5030 with software Esprit 1.9 (Bruker, Germany). The SEM images were recorded with the four-quadrants backscattered electron detector (BSD signal) at 15 kV.

### X-ray diffraction

X-ray diffraction (XRD) patterns for determination of the phase assemblage were recorded for EAC-1A lunar regolith simulant powders and sintered materials. Before the measurements, the specimens were manually ground with mortar and pestle (agate) and filled into specimen holders by top-loading. XRD patterns were recorded in Bragg–Brentano geometry on a laboratory diffractometer (Ultima IV, Rigaku) at room temperature under the following conditions: CuKα radiation (*λ* = 1.541874 Å); tube operating at 40 kV, 40 mA; sampling interval: 0.02° 2*θ*; scan rate: 0.5° 2*θ* min^−1^; scanning range: 5°–65° 2*θ*; divergence slit: in-plane 1/2°, axial 10 mm; strip detector D/teX Ultra with 5° Soller slits. Phase identification was done using the PDF-4+2023 database.

For Rietveld quantitative phase analysis (RQPA) of EAC-1A, the specimen was first manually ground to particle sizes < 500 µm. Subsequently, the resulting powder was ground in a McCrone ‘micronizing’ mill in propan-2-ol for 5 min. One batch was ground without any addition, and the other batch was ground together with 10% zincite (ZnO; AppliChem, grade p.a., min. 99.5%). The resulting slurries were dried at 40 °C overnight. The resulting specimen powders were filled into the specimen holder for the XRD measurements by side-loading. XRD patterns for RQPA were recorded in Bragg–Brentano geometry on the same diffractometer as above at room temperature under the following conditions: CuKα radiation (*λ* = 1.541874 Å); tube operating at 40 kV, 40 mA; sampling interval: 0.01° 2*θ*; scan rate: 0.2° 2*θ* min^−1^; scanning range: 5°–120° 2*θ*; divergence slit: in-plane 1/6°, axial 10 mm; strip detector D/teX Ultra with 5° Soller slits. The NIST Standard Reference Material 660c (LaB_6_) was employed to determine the instrumental parameters of the diffractometer. RQPA was performed with TOPAS version 5, using crystal structure data files from the ICSD; the specimens with and without addition of zincite as internal standard yielded similar results.

### Karl-Fischer titration

The water content of the sieved and unsieved specimens of the regolith simulant EAC-1A was determined by Karl-Fischer titration technique. The equipment used was a 874 Oven specimen processor (Metrohm) and KF Titrator Coulometric (Metrohm). For the experiments, the oven temperature was 180 °C and the gas flow 40 mL.

### Thermogravimetric analysis and differential scanning calorimetry

Simultaneous thermogravimetric (TGA) and differential scanning calorimetry (DSC) analyses were performed on a TGA/DSC 3+ STARe device (Mettler Toledo). A specimen powder mass of ~ 10 mg was heated in an Al_2_O_3_ crucible to ~ 1350 °C at a heating rate of 10 °C/min under flowing nitrogen and then cooled to room temperature at the same rate. The nitrogen flux during the measurement was 80 mL/min.

### Infrared spectroscopy

Attenuated total reflection-Fourier transform infrared (ATR-FTIR) spectra of the specimen powders were recorded at room temperature on a mid-IR spectrometer (Nicolet iS 50 FTIR, Thermo Scientific) in the wavenumber range 4000–400 cm^−1^ with an optical resolution of 0.125 cm^−1^. Three measurements were done; for each measurement, 32 scans were recorded and averaged. The measurements were highly reproducible; thus, only one of the three spectra is shown in the “Results” section.

### X-ray fluorescence analysis

The chemical composition of the unsieved simulant was measured after drying and after thermal treatment at 900 °C (1 h in vacuum) by X-ray fluorescence (Panalytical MagixPro, Malvern Panalytical GmbH, Germany) and analyzed with the FLX-RAW-Pro database.

### Hot stage microscopy

Thermal analysis of the simulant was carried out using HSM (EM301, Hesse Instruments). HSM is a thermal analysis technique, in which optical technologies are used in order to correlate the shape of the molten specimen and its corresponding temperature. Therefore, the changes produced in the silhouette of the specimens when they are exposed to changes in temperature are examined.

In these experiments, the powder was pressed in a mold and the result was a cylindrical specimen. During heating, a CCD camera records snapshots of the backscattered specimen’s outline. The recorded images are then analysed for the determination of the characteristic temperatures (DT, ST, HT, FT). This task is performed by the instrument software EMI III, version 3.1.6-0 (in accordance with DIN 51730:1984, as implemented in the software).

For the start of sintering temperature (SST), the value was determined manually, as there is no standard procedure that can be set in the microscope’s software. As such, the SST was determined as follows: two adjutant lines fitted to the obtained sintering graph, which displays the specimen area (px^2^) and specimen temperature (°C). The first one is tangent to the lineal thermal expansion of the curve and the second one, tangent to the shrinkage part of the curve. The intersection of the two lines was considered as the sintering temperature in this study (see Supplementary Fig. S9 online).

In addition, the furnace of the hot stage microscope is equipped with a gas circulation system which allows the performance of the experiments under different atmosphere compositions. In this case, the system was used to pump argon in the furnace. First, the HSM experiments were performed under air. In a second stage, the same experiments were repeated pumping argon in the microscope’s furnace, in order to analyse the effect of the absence of air on the results obtained. In the experiments performed with argon, the gas flow was 3L/h according to the information stated in DIN 51730.

Simulant specimens with three particle size fractions were analysed: as received simulant, 500 µm > *P* > 100 µm and *P* < 100 µm. In these cases, a small amount of material is pressed to form a specimen analysable by the HSM software. The fraction larger than 500 µm was considered to be too coarse to be pressed and conform a specimen. Each experiment was performed three times.

### Pressing and sintering of EAC-1A lunar regolith simulant

Tablets were pressed in a hydraulic press (Paul-Otto Weber GmbH, Germany) with a 16 mm diameter round die. The pressing was done in 2 steps: 10 s with a 5 kN force (6 MPa pressure) and 20 s with a 25 kN force (31 MPa pressure). No binder was used except for the unsieved/dry powder, for which a drop of isopropanol had to be added to obtain stable tablets. For sintering, the heating rate was set to 10 °C/min and the holding time 1 h. Cooling lasted 1 h to reach 20 °C. The sintering experiments were carried out in an evacuated Nabertherm horizontal tube oven (RHTC-80 450). The pressed tables were placed on top of a flat alumina foam plate and enclosed between two alumina crucibles to improve the heat distribution homogeneity. The crucible was then carefully placed in the middle of the tube oven. This type of oven typically has a length of approximately 210 mm around the center of the tube in which the temperature is constant within ± 5 °C.

The geometrical density was measured for 10 tablets of each group and averaged. The diameter and thickness of each tablet was measured with a digital calliper (± 0.01 mm) and with a micrometer screw gauge (± 0.001 mm) respectively. The mass was measured on a Sartorius laboratory scale BCE2202:1S (± 0.01 g).

The bulk density of the sintered tablets was measured by Archimede’s method according to ISO 18754:2020 in deionized water (boiling method). The geometrical density was also determined by measurement of geometric dimensions and mass and followed the same trend, with a deviation within ± 5% from the density measured by Archimede’s method. The true density was measured by helium pycnometry (Pycnomatic ATC, Porotec).

### Ball-on-three-balls mechanical tests

The ball-on-three-balls method measures the load applied on the tablet until it breaks. The fracture load F (N) is then used to calculate the biaxial fracture stress σ_max_ (MPa):

$${\sigma }_{max}=\frac{F}{{t}^{2}}f\left(x,z\right); x=\frac{t}{R}; z=\frac{{R}_{a}}{R}; {R}_{a}=\frac{2\sqrt{3}{R}_{b}}{3}$$

where *t* is the thickness of the specimen (in mm); *f* is a scale function that is calculated numerically, see reference^34^; *R*_*a*_ the radius of the support (in mm), *R*_*b*_ the radius of the balls (in mm), *R* the radius of the specimen (in mm). For each group, ten tablets with a diameter of approximately 15.5 mm and a thickness of approximately 2.5 mm were measured. The diameter and thickness of each tablet was measured with a digital calliper (± 0.01 mm) and with a micrometer screw gauge (± 0.001 mm) respectively.

### Supplementary Information

Supplementary Information.

## Data Availability

The HSM datasets are available from the *figshare* repository, https://figshare.com/projects/Material_aspects_of_sintering_of_EAC-1A_lunar_regolith_simulant/166898. The other datasets used and/or analyzed during the current study are available from the corresponding authors on reasonable request.
