# Supplementary material for: Material aspects of sintering of EAC-1A lunar regolith simulant
Source: Sci Rep. 2023 Dec 27;13:23053. doi: 10.1038/s41598-023-50391-y (PMC10754926; doi:10.1038/s41598-023-50391-y)
Supplement: Supplementary file 1 — Supplementary Information. [file 41598_2023_50391_MOESM1_ESM.docx]

**Supplementary Information**

for

**Material aspects of sintering of EAC-1A lunar regolith simulant**

**Juan-Carlos Ginés-Palomares^1^*, Miranda Fateri^1^, Tim Schubert^2^, Lilou de Peindray d'Ambelle^3^, Sebastian Simon^4^, Gregor J. G. Gluth^4^, Jens Günster^3^, Andrea Zocca^3^***

^1^ Faculty of Mechanical Engineering and Materials Science, Aalen University, Beethovenstraße. 1, 73430 Aalen, Germany.

^2^ Materials Research Institute Aalen, Aalen University, Beethovenstraße. 1, 73430 Aalen, Germany.

^3^ Division 5.4 Advanced Multi-materials Processing, Bundesanstalt für Materialforschung und -prüfung (BAM), Unter den Eichen 87, 12205 Berlin, Germany

^4^ Division 7.4 Technology of Construction Materials, Bundesanstalt für Materialforschung und -prüfung (BAM), Unter den Eichen 87, 12205 Berlin, Germany

**X-ray diffraction**

**
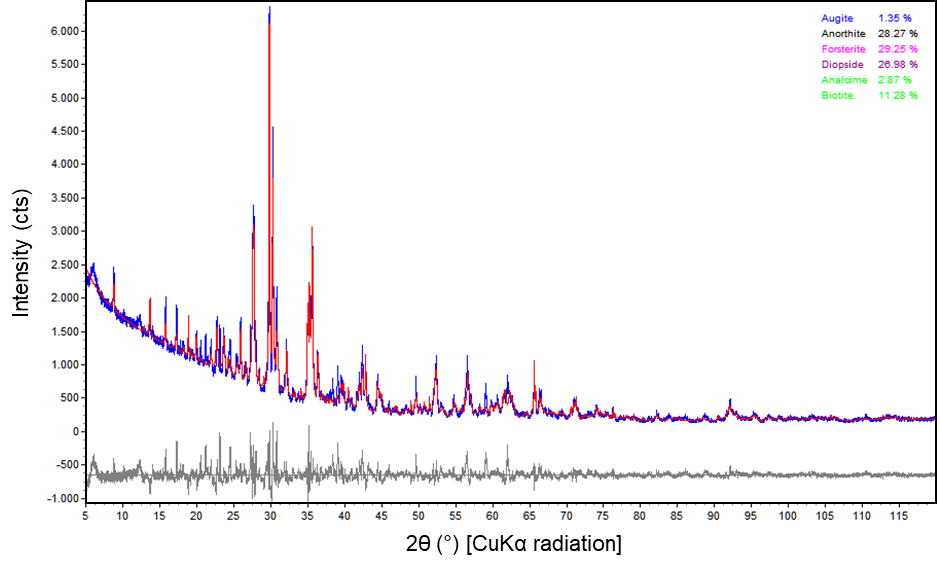
**

Supplementary Figure S1. XRD pattern for Rietveld quantitative phase analysis (RQPA) of EAC-1A.

**X-ray fluorescence analysis**

|  |  | **EAC-1A 900°C in vacuum (24 h)** | **EAC-1A as-received** |
| --- | --- | --- | --- |
|  |  |  |  |
| Sum | (%) | 99.61 | 96.60 |
| Al_2_O_3_ | (%) | 11.53 | 11.01 |
| BaO | (%) | 0.02 | 0.03 |
| CaO | (%) | 10.45 | 9.93 |
| Cr_2_O_3_ | (%) | 0.06 | 0.06 |
| CuO | (%) | 0.01 | 0.01 |
| Fe_2_O_3_ | (%) | 12.68 | 12.37 |
| HfO_2_ | (%) | 0.02 | 0.02 |
| K_2_O | (%) | 0.99 | 0.98 |
| MgO | (%) | 14.05 | 14.28 |
| Mn_2_O_3_ | (%) | 0.23 | 0.23 |
| Na_2_O | (%) | 2.76 | 2.79 |
| NiO | (%) | 0.05 | 0.05 |
| P_2_O_5_ | (%) | 0.64 | 0.63 |
| PbO | (%) | 0.00 | 0.00 |
| SiO_2_ | (%) | 43.72 | 41.89 |
| SO_3_ | (%) | 0.05 | 0.09 |
| SrO | (%) | 0.12 | 0.13 |
| TiO_2_ | (%) | 2.19 | 2.09 |
| V_2_O_5_ | (%) | 0.02 | 0.02 |
| ZnO | (%) | 0.01 | 0.02 |
| ZrO_2_ | (%) | 0.00 | 0.00 |

*Supplementary Table S1. XRF composition of EAC-1A as-received and after 24 h heat treatment at 900°C in vacuum. The composition is expressed in terms of equivalent oxides.*

**Hot stage microscopy**


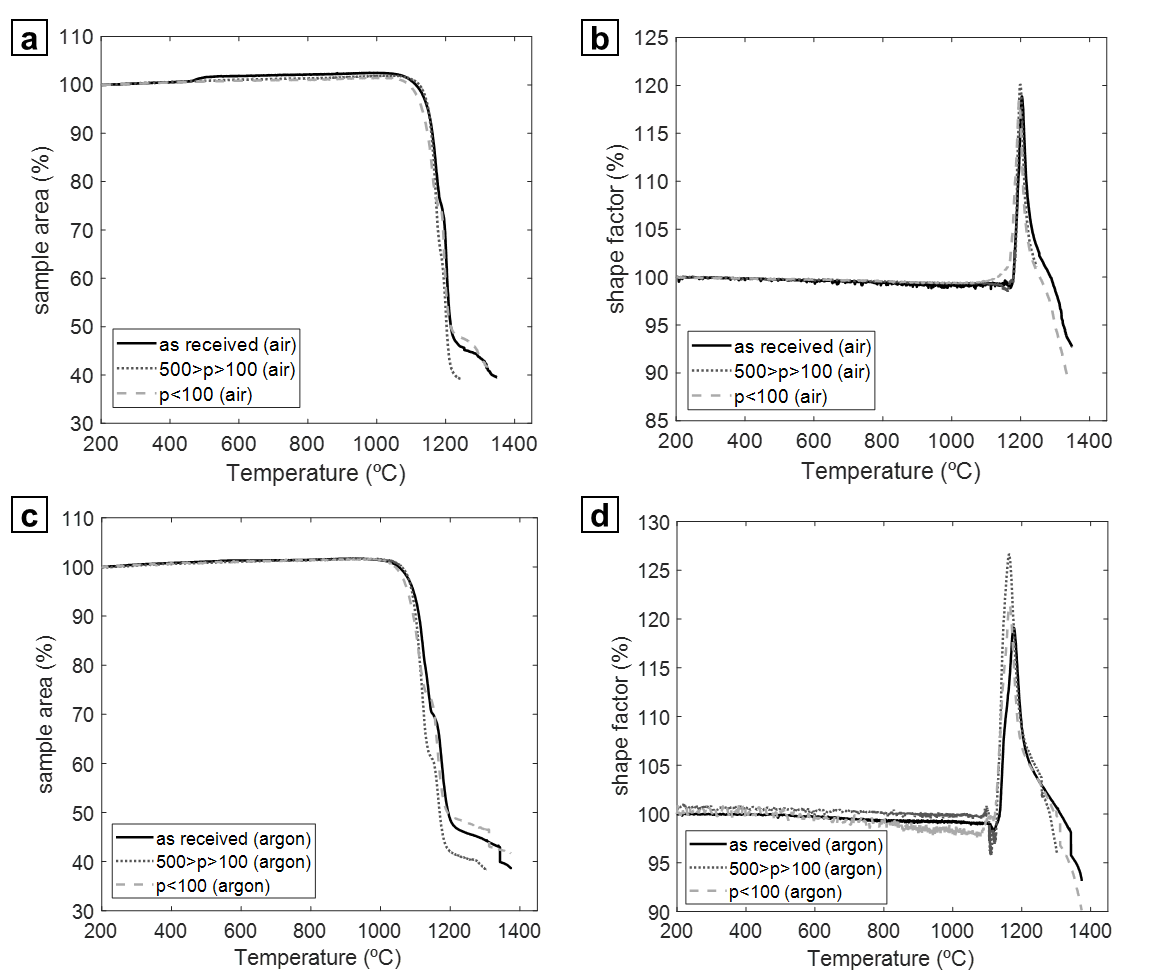


Supplementary Figure S2. Results of the Hot stage microscope experiments. a: sample area of the experiments carried out in air. b: shape factor of the experiments carried out in air. c: sample area of the experiments carried out in argon. d: shape factor of the experiments carried out in argon.

**Sintering of EAC-1A lunar regolith simulant**


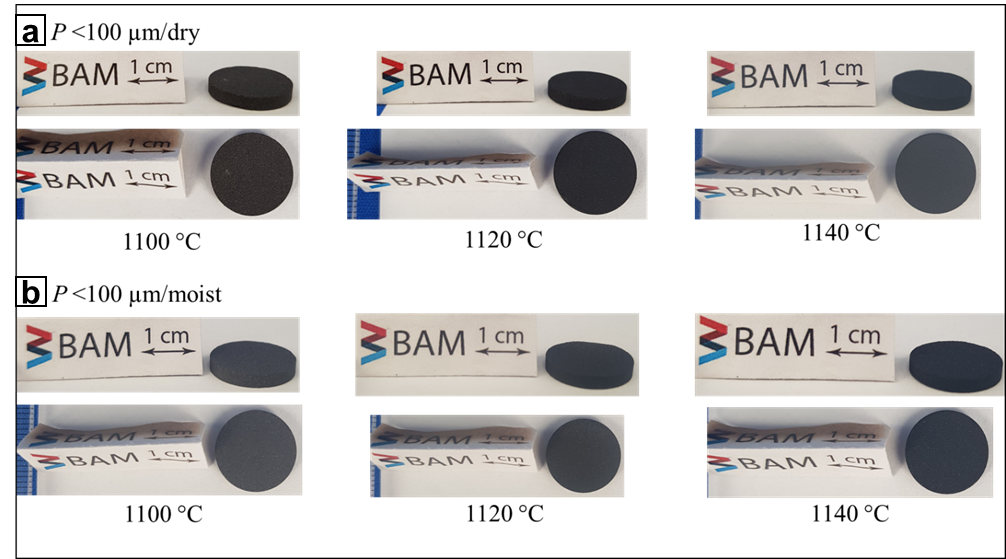


Supplementary Figure S3. EAC-1A tablets from pressed P < 100 µm powder after sintering. (a) dry samples (b) moist samples.


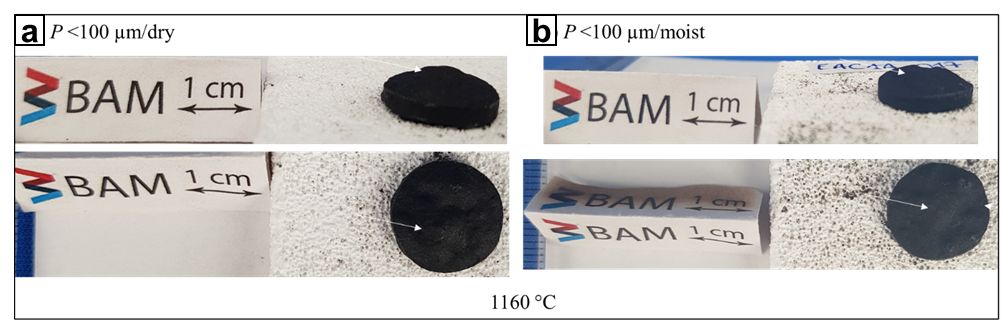


Supplementary Figure S4. EAC-1A tablets from pressed P < 100 µm powder after sintering at 1160 °C. (a) dry sample (b) moist sample.


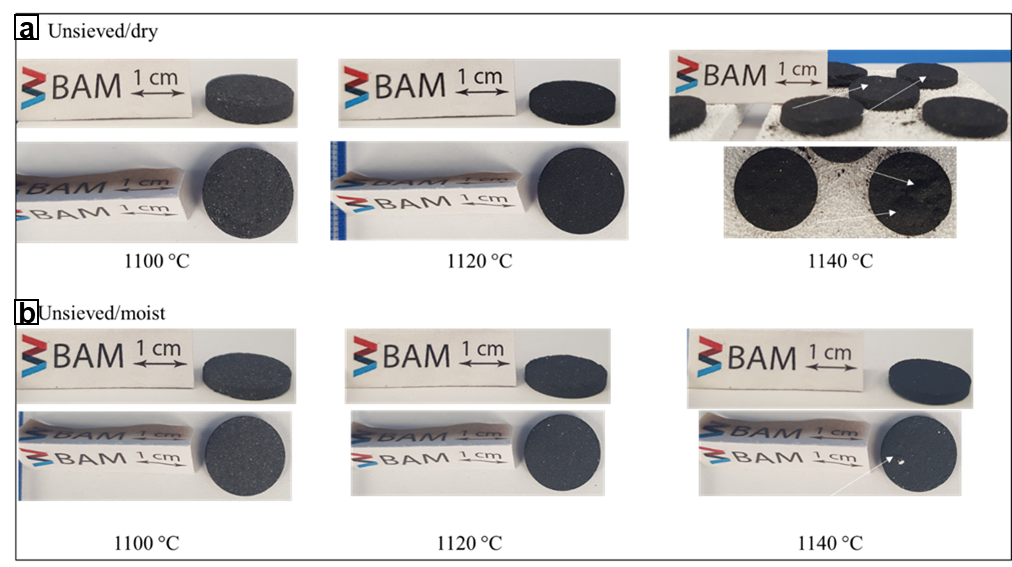


Supplementary Figure S5. EAC-1A tablets from pressed unsieved powder after sintering. (a) dry samples (b) moist samples.


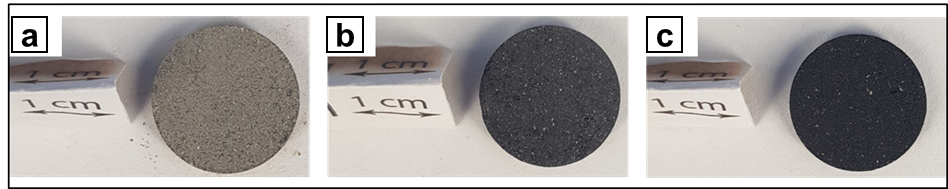


Supplementary Figure S6. EAC-1A tablets from pressed unsieved/dry powder- a) after pressing ; b) after sintering at 1100 °C ; c) after sintering at 1120 °C.

**SEM-EDS Analysis**

Supplementary Figure S7. EDS spectra and phase assignment relative to the SEM microstructure analysis in Figure 6 of the manuscript.

**Mechanical characterization**

Supplementary Figure S8. Load-displacement curves of EAC-1A tablets tested by ball-on-three-balls biaxial test.

The fracture load F (N) is used to calculate the biaxial fracture stress σ_max_ (MPa):

$$\sigma_{max}=\frac{F}{t^{2}}f\left( x,z \right) x=\frac{t}{R} z=\frac{R_{a}}{R} R_{a}=\frac{2\sqrt{3}R_{b}}{3}$$

where t is the thickness of the sample (in mm); f is a scale function that is calculated numerically, see reference [34]; Ra the radius of the support (in mm), Rb the radius of the balls (in mm), R the radius of the sample (in mm).

**Methods. Hot Stage Microscopy**


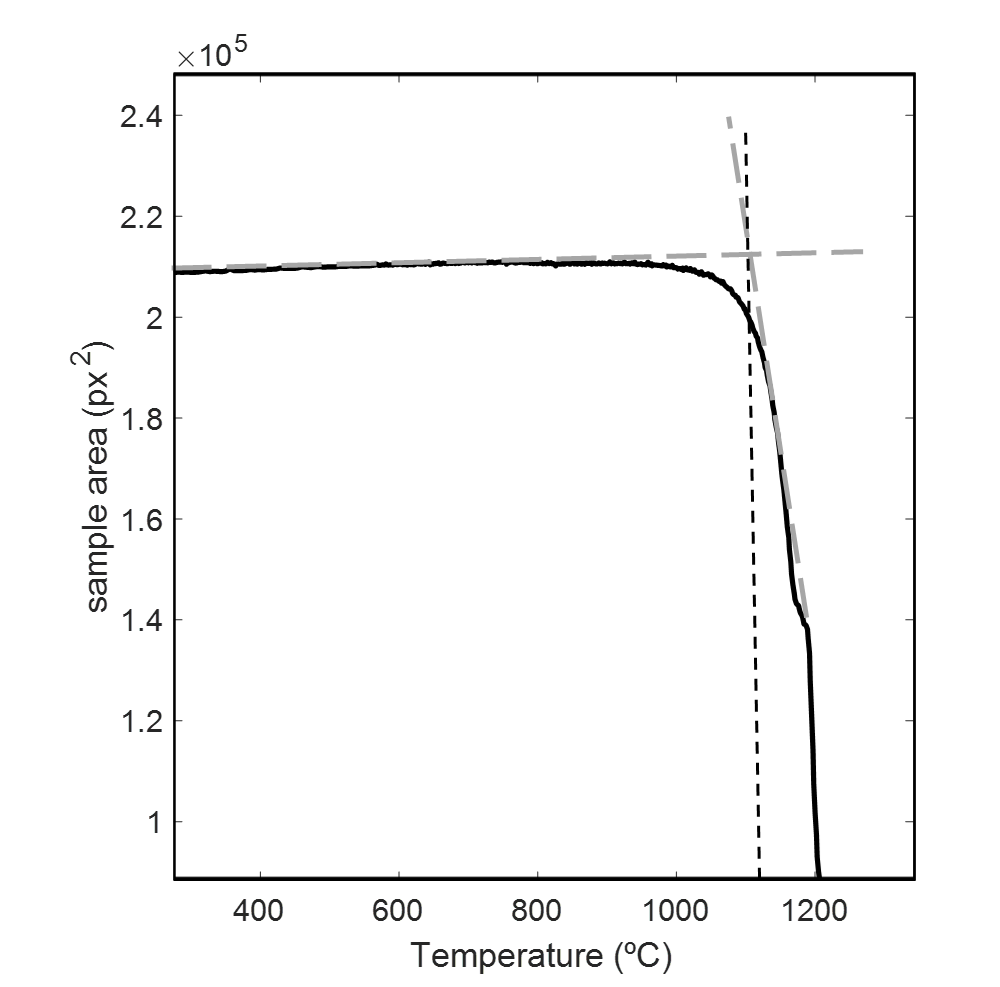


Supplementary Figure S9. Example of Start of Sintering Temperature determination.
